# Bacterial extracellular vesicles as central mediators of microbiota–host communication

**DOI:** 10.3389/fmicb.2026.1910885

**Published:** 2026-09-18

**Authors:** Sinem Oktem Okullu, Sümeyye Akcelik-Deveci, Yaren Buyukcolak-Cebeci, Selcen Necibe Gokduman, Gizem Sele

**Affiliations:** 1 Department of Medical Microbiology, School of Medicine, Acibadem Mehmet Ali Aydinlar University, Istanbul, Türkiye; 2 Department of Molecular Biology and Genetics, Faculty of Sciences, Boğaziçi University, Istanbul, Türkiye; 3 Department of Medical Biotechnology, Institute of Health and Science, Acibadem Mehmet Ali Aydinlar University, Istanbul, Türkiye

**Keywords:** gut microbiota, innate immunity, outer membrane vesicles (OMVs), postbiotics, quorum sensing

## Abstract

Bacterial extracellular vesicles (BEVs) are nanoscale, lipid bilayer–enclosed structures released by both Gram-negative and Gram-positive bacteria that act as active mediators of microbial and host–microbe communication rather than simple byproducts of membrane turnover. By packaging proteins, nucleic acids, lipids, and metabolites, BEVs participate in quorum sensing, biofilm regulation, horizontal gene transfer, and immune modulation, with functional outcomes that depend strongly on microbial origin and environmental context: pathogen-derived BEVs frequently promote inflammation and tissue damage, whereas commensal- and probiotic-derived BEVs are more often linked to epithelial protection and immune regulation. These properties have positioned BEVs as promising translational tools for biomarker discovery, vaccine development, and engineered drug delivery. However, vesicle heterogeneity, inconsistent isolation and purification methods, and an incomplete understanding of *in vivo* behavior continue to limit reproducibility and slow clinical translation. Here, we synthesize current evidence on BEV biogenesis, cargo selection, and biological function, and outline the methodological and translational challenges that must be addressed to establish BEVs as a validated interface between microbial activity and host physiology.

## 1 Introduction

Bacterial extracellular vesicles (BEVs) are nanosized lipid bilayer structures secreted by a broad range of Gram-negative and Gram-positive bacteria. They were initially regarded as byproducts of bacterial envelope turnover or metabolic waste. However, accumulating evidence indicates that BEVs participate in microbial communication, environmental adaptation, and host–microbe interactions (De Langhe et al., 2024). BEVs contain a wide range of cargo molecules, including proteins, lipids, enzymes, metabolites, and nucleic acids such as DNA and RNA, as well as microbe-associated molecular patterns (MAMPs). Vesicle encapsulation protects these components from extracellular degradation and facilitates their transfer between microbial and host cells (De Langhe et al., 2024; Qian et al., 2025).

Interest in BEVs has increased substantially over the past decade, partly due to advances in imaging technologies, multi-omics approaches, and vesicle isolation methods. A recent large-scale mapping study highlighted the rapid expansion of the field but also emphasized persistent variability in isolation and characterization workflows, which continues to complicate comparisons between studies (De Langhe et al., 2024). Earlier work focused mainly on BEVs derived from pathogenic bacteria. More recent studies, however, have expanded this perspective to include their roles in commensalism, immune regulation, and microbiota–host communication (Qian et al., 2025; Melo-Marques et al., 2024; Yi et al., 2025).

BEVs enable bacteria to transport signaling molecules, enzymes, and other cargo beyond direct cell-to-cell interactions. Within microbial communities, they contribute to nutrient exchange, biofilm organization, and interbacterial communication. Several studies also indicate that BEVs can carry quorum-sensing (QS) molecules. Encapsulation of autoinducers within vesicles may reduce signal dilution and protect signaling molecules from enzymatic degradation, helping maintain coordinated microbial behavior under changing environmental conditions (Mashburn and Whiteley, 2005).

BEVs also interact extensively with host tissues. Their small size facilitates diffusion across mucosal barriers and promotes interactions with epithelial and immune cells, where they can activate pattern-recognition receptors (PRRs) and influence innate and adaptive immune responses (Qian et al., 2025; Melo-Marques et al., 2024; Yi et al., 2025). In the gastrointestinal tract, microbiota-derived BEVs have been associated with epithelial barrier maintenance, intestinal homeostasis, and signaling along extraintestinal axes. Recent studies further suggest roles in hepatic metabolism, systemic inflammation, and tumor-associated processes (Qian et al., 2025; Yi et al., 2025; Lin et al., 2025; Li Y. et al., 2026; Verbunt et al., 2024). Importantly, these effects are not restricted to pathogenic bacteria. Vesicles derived from commensal and probiotic organisms can also exert anti-inflammatory and immunomodulatory effects, indicating that BEVs may contribute to both physiological homeostasis and disease progression depending on their microbial origin and molecular cargo (Melo-Marques et al., 2024; Zhang et al., 2025; Hu et al., 2021).

The translational potential of BEVs has attracted increasing attention in recent years. Because of their stability and modifiable cargo composition, they are being investigated as potential biomarkers, vaccine platforms, and drug delivery systems within the broader framework of postbiotic therapeutics (De Langhe et al., 2024; Qian et al., 2025). However, several limitations still hinder clinical translation, including vesicle heterogeneity, scalability issues, and the lack of standardized isolation and characterization protocols (De Langhe et al., 2024; Welsh et al., 2024). Addressing these challenges will be important for improving both mechanistic studies and future therapeutic applications.

In this review, we summarize current knowledge regarding BEV biogenesis, cargo composition, and biological functions. We also discuss their roles in microbial ecology, host immune regulation, and disease, together with current methodological and translational challenges in the field (Table 1 and Figure 1).

**Table 1:** Representative studies investigating bacterial extracellular vesicles (BEVs) in microbiota–host communication and disease.

Study (Year)	BEV source/Model	Experimental system	Main finding	Biological significance	Isolation/ characterization method
Melo-Marques et al. (2024)	Gut microbiota-derived BEVs	Review/Immunology	BEVs regulate host immunity through epithelial and immune-cell interactions	Established BEVs as key mediators of microbiota–host communication	Review (not applicable)
Nie et al. (2025)	*Bifidobacterium longum* NSP001 BEVs	Murine colitis model	BEVs alleviated colitis and improved barrier integrity	Demonstrated postbiotic therapeutic potential of probiotic-derived BEVs	0.45 μm filtration; ultracentrifugation (150,000 × g, 2 h, 4 °C); TEM
Han et al. (2024)	*Escherichia coli* Nissle 1917 OMVs	Experimental colitis	Enhanced epithelial barrier function and reduced inflammation	Supported use of engineered probiotic vesicles in intestinal disorders	Ultracentrifugation (150,000 × g); microsphere-coupled formulation
Preet et al. (2025)	*Bifidobacterium*-derived BEVs	Lung cancer + anti-PD-1 model	Improved immunotherapy response via immune modulation	Linked microbiota-derived BEVs to cancer immunotherapy	Standard bacterial EV ultracentrifugation protocol; NTA/TEM
Zheng et al. (2024)	*Fusobacterium nucleatum* BEVs	Colorectal cancer tissues	Promoted bacterial adhesion and persistence within tumors	Identified BEVs as contributors to tumor microenvironment remodeling	Differential centrifugation; density-gradient ultracentrifugation
Kawagishi et al. (2025)	*Escherichia coli* BEVs	Interspecies bacterial interaction model	Suppressed growth and virulence of Group A Streptococcus	Revealed BEVs as mediators of interspecies competition	Ultracentrifugation-based EV isolation; TEM
Xu et al. (2026)	Environmental bacterial BEVs	Aquatic microbial communities	Vesicle-associated quorum-sensing molecules enhanced signal delivery	Demonstrated BEVs as structured communication vectors	Sequential filtration (3–0.2 μm) + TFF concentration; ultracentrifugation (100,000 × g); OptiPrep density-gradient purification; NanoFCM, TEM, zeta potential
Wen and Herman (2024)	Multiple bacterial species	Review/Molecular microbiology	BEVs transfer DNA, RNA and proteins between cells	Highlighted their role in horizontal gene transfer and AMR dissemination	Review (not applicable)
Lin et al. (2025)	Gut microbial BEVs	Atherosclerosis-related models	BEVs promoted vascular inflammation and plaque progression	Connected BEVs with cardiometabolic disease	Review (not applicable)
Qian et al. (2025)	Gut microbiota-derived BEVs	Review/Translational research	BEVs possess diagnostic and drug-delivery potential	Positioned BEVs as emerging nanomedicine platforms	Review (not applicable)

**Figure 1:**
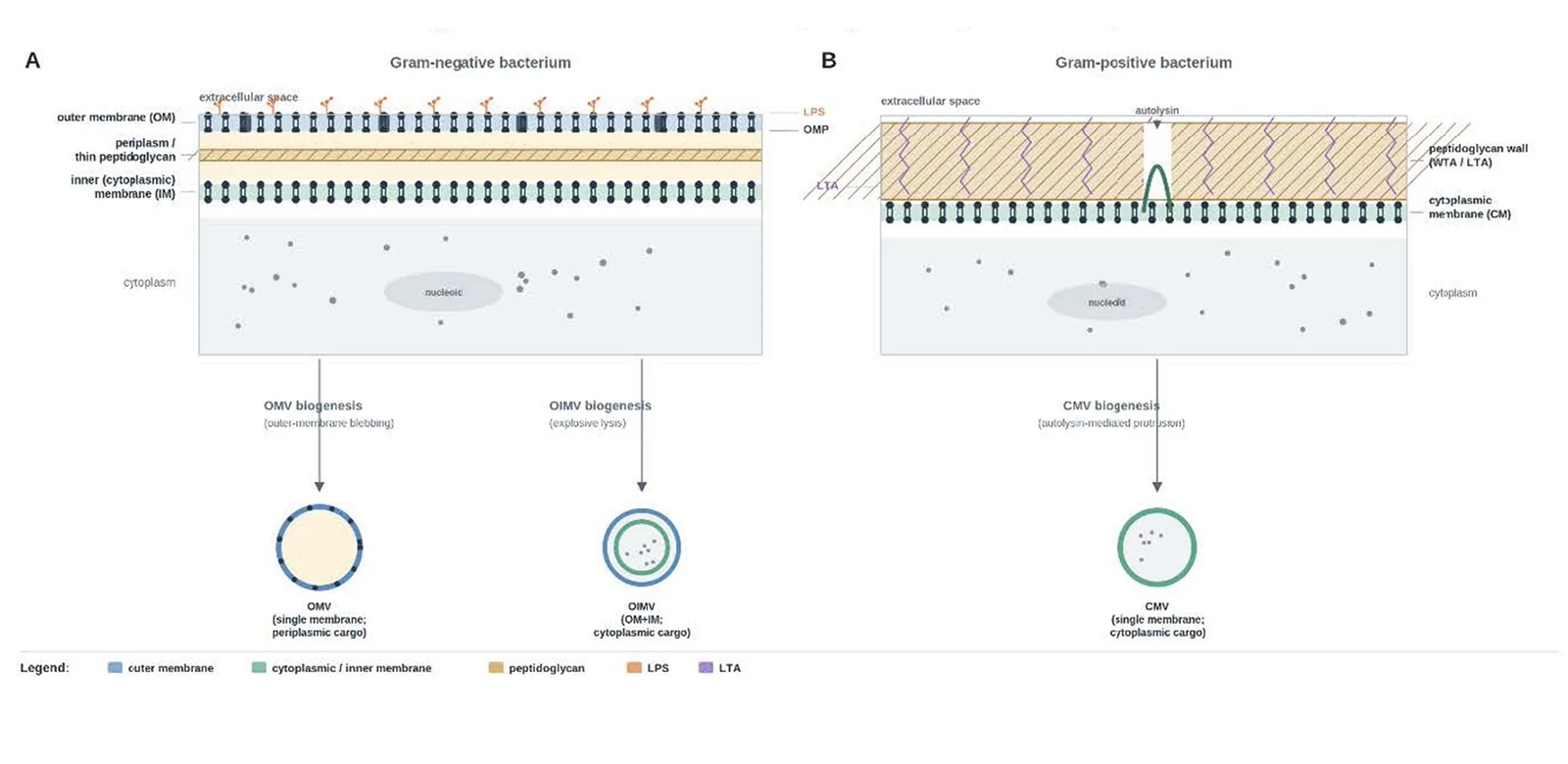
Biogenesis of bacterial extracellular vesicles (BEVs) in Gram-negative and Gram-positive bacteria. Schematic comparison of BEV biogenesis pathways in Gram-negative and Gram-positive bacteria, redrawn to reflect the correct envelope topology. In Gram-negative bacteria **(A)**, the outer membrane lies external to a thin peptidoglycan layer within the periplasm, which in turn overlies the inner (cytoplasmic) membrane; classical outer membrane vesicles (OMVs) form by outer-membrane blebbing and carry periplasmic cargo, whereas outer–inner membrane vesicles (OIMVs) arise through explosive-lysis-associated mechanisms and additionally contain cytoplasmic material. In Gram-positive bacteria **(B)**, a thick peptidoglycan wall lies external to a single cytoplasmic membrane; cytoplasmic membrane vesicles (CMVs) form when localized, autolysin-mediated peptidoglycan remodeling creates a gap through which the membrane protrudes and pinches off under turgor pressure (De Langhe et al., 2024; Schwechheimer and Kuehn, 2015; Kulp and Kuehn, 2010; Jeong et al., 2022; Zou et al., 2025).

## 2 Biogenesis and cargo selection of bacterial extracellular vesicles

The biological functions of bacterial extracellular vesicles (BEVs) are closely associated with their mechanisms of formation and cargo composition. BEVs are not a uniform population of particles; instead, they comprise heterogeneous membrane-derived structures generated through multiple biogenetic pathways. Different modes of vesicle formation can produce vesicles with distinct membrane compositions, cargo profiles, and biological activities (De Langhe et al., 2024). This heterogeneity is likely to influence how BEVs interact with surrounding microbial communities and host tissues (Figure 2).

**Figure 2:**
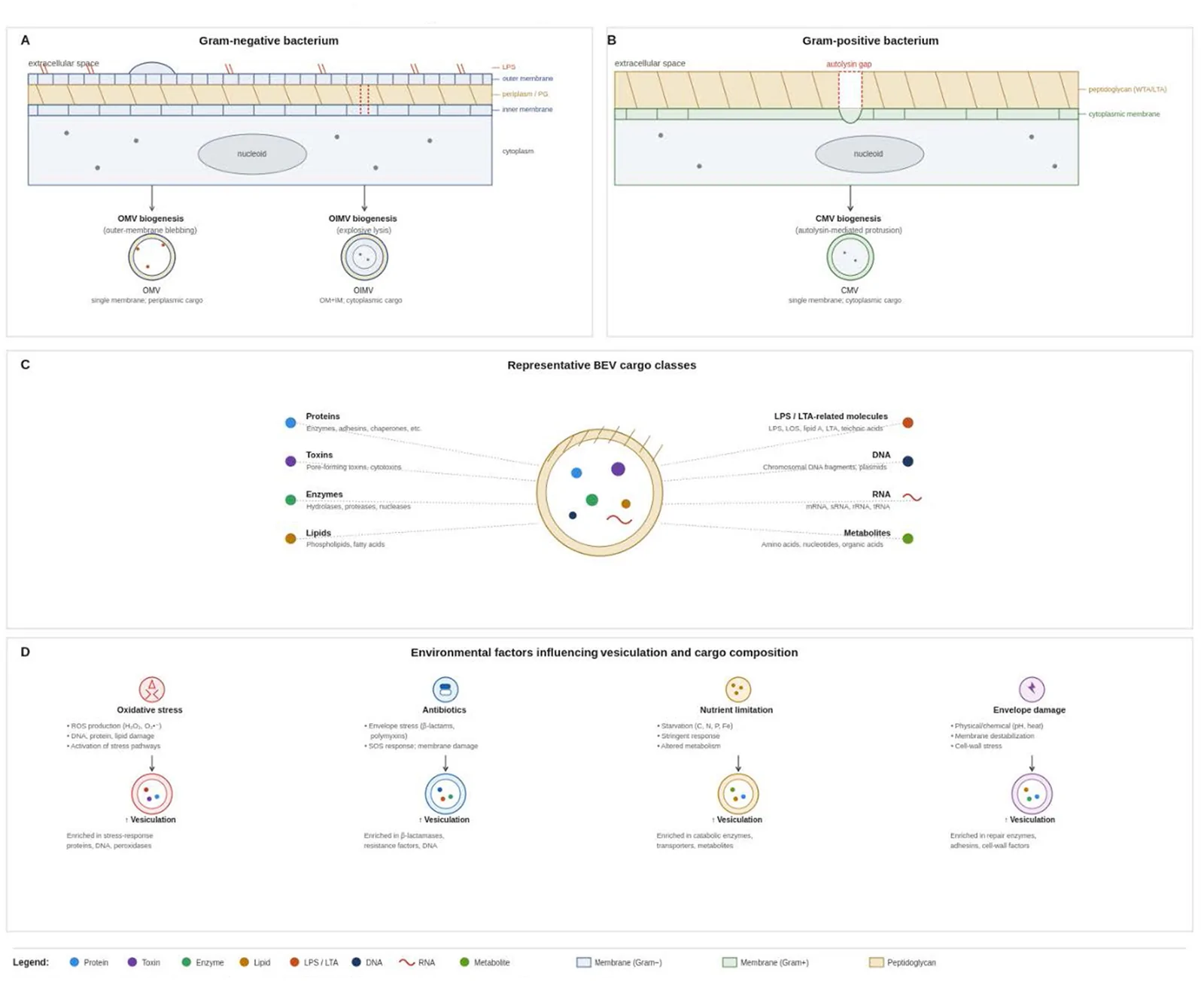
Biogenesis and cargo selection of bacterial extracellular vesicles. Schematic overview of bacterial extracellular vesicle (BEV) biogenesis in Gram-negative and Gram-positive bacteria, highlighting major formation pathways and key determinants of cargo composition. Environmental conditions influencing vesiculation and molecular loading are also indicated (Schwechheimer and Kuehn, 2015; Caruana and Walper, 2020; Toyofuku et al., 2023; Kulp and Kuehn, 2010; Tsering et al., 2024; Roier et al., 2016; Orench-Rivera and Kuehn, 2021). **(A)** Outer membrane vesicle (OMV) and outer-inner membrane vesicle (OIMV) biogenesis in a Gram-negative bacterium. **(B)** Cytoplasmic membrane vesicle (CMV) biogenesis in a Gram-positive bacterium. **(C)** Representative BEV cargo classes, including proteins, toxins, enzymes, lipids, LPS/LTA, DNA, RNA, and metabolites. **(D)** Environmental factors (oxidative stress, antibiotics, nutrient limitation, and envelope damage) that influence vesiculation and cargo composition.

### 2.1 Biogenesis in gram-negative and gram-positive bacteria

In Gram-negative (diderm) bacteria, vesicle formation has classically been linked to outward budding of the outer membrane, resulting in the release of outer membrane vesicles (OMVs). Several mechanisms have been proposed to contribute to this process, including localized dissociation of the outer membrane from the underlying peptidoglycan layer, phospholipid redistribution, accumulation of misfolded periplasmic proteins, and activation of envelope stress pathways (Schwechheimer and Kuehn, 2015; Kulp and Kuehn, 2010).

More recent studies suggest that vesicle formation can also occur through lysis-associated mechanisms. Processes such as explosive cell lysis and bubbling cell death generate membrane-derived particles containing components of both the inner and outer bacterial membranes together with cytoplasmic material (De Langhe et al., 2024; Kulp and Kuehn, 2010). These mechanisms may partly explain why some BEV preparations contain intracellular molecules, including nucleic acids and ATP-associated metabolites.

In Gram-positive (monoderm) bacteria, vesicle biogenesis is less straightforward because these organisms lack an outer membrane and possess a thick peptidoglycan layer. Vesicle production was therefore initially considered unlikely. However, multiple studies now confirm that Gram-positive bacteria can actively release extracellular vesicles. Current models suggest that localized cell wall remodeling by autolysins and related peptidoglycan-modifying enzymes permits membrane protrusion under turgor pressure, enabling vesicles to pass through the cell envelope (De Langhe et al., 2024; Jeong et al., 2022; Zou et al., 2025). These findings indicate that vesicle-mediated secretion is not restricted to a particular bacterial envelope structure and may represent a conserved feature across diverse bacterial groups (Schwechheimer and Kuehn, 2015; Kulp and Kuehn, 2010).

Mycobacteria occupy a structural niche that maps onto neither the Gram-negative nor the Gram-positive biogenesis models described above, and their inclusion illustrates that BEV biogenesis diversity extends beyond the classical diderm/monoderm dichotomy. The mycobacterial cell envelope comprises a peptidoglycan layer covalently linked to arabinogalactan, which is in turn esterified to exceptionally long-chain (up to C60) mycolic acids that intercalate with additional free lipids to form an outer, mycolic-acid-rich bilayer known as the mycomembrane, external to which a loosely associated capsule of polysaccharides, proteins, and lipids is often present. Because of this multilayered envelope, both the route by which mycobacterial extracellular vesicles (MEVs) cross the cell wall and their membrane of origin have been difficult to resolve. Contrary to an initial expectation that MEVs would arise from mycomembrane blebbing, analogous to Gram-negative OMV formation, lipidomic analyses of *Mycobacterium tuberculosis* MEVs produced under standard growth conditions show enrichment in polar phospholipids characteristic of the cytoplasmic membrane rather than in mycomembrane-specific mycolic acid species, indicating that the cytoplasmic membrane, not the mycomembrane, is the primary source under these conditions. MEV release in *M. tuberculosis* is regulated by environmental cues, most notably iron limitation, and requires dedicated molecular machinery, including the bacterial dynamin-like proteins IniA and IniC, which are induced as part of a cell-envelope stress response and are required for efficient vesicle biogenesis both in axenic culture and during macrophage infection. This contrasts with the related mycolic-acid-containing organism *Corynebacterium glutamicum*, in which outer mycomembrane blebbing does generate a distinct, mycolic-acid-enriched vesicle population, indicating that even among mycolic-acid-containing bacteria the dominant biogenesis route, and consequently vesicle composition, is genus- and condition-specific rather than conserved. Given the central role of *M. tuberculosis* in human disease and the demonstrated immunomodulatory and vaccine-relevant properties of MEVs, mycobacteria represent a mechanistically distinct addition to the biogenesis framework outlined above rather than a simple variant of Gram-positive vesiculation (Salgueiro et al., 2024).

### 2.2 Determinants of selective cargo loading

BEV cargo composition does not simply mirror the surrounding membrane or cytoplasmic content. Proteomic and lipidomic analyses have repeatedly shown enrichment of specific outer membrane proteins, adhesins, toxins, and stress-associated factors relative to their abundance in the parent cell (Schwechheimer and Kuehn, 2015; Caruana and Walper, 2020; Toyofuku et al., 2023). These findings suggest that cargo incorporation is at least partly regulated rather than entirely stochastic.

The presence of nucleic acids within BEVs has also attracted considerable interest. Vesicle encapsulation protects DNA and RNA from extracellular nuclease activity, improving their stability outside the cell. This may facilitate horizontal gene transfer and intercellular signaling after uptake by recipient bacteria or host cells (Schwechheimer and Kuehn, 2015; Tsering et al., 2024).

A growing body of work has begun to resolve the molecular determinants that govern which membrane components are selectively incorporated into BEVs, rather than treating cargo selection as a uniform or stochastic process. At the level of lipid composition, the VacJ/Yrb ATP-binding cassette transporter (also referred to as the Mla pathway) maintains phospholipid asymmetry across the outer membrane by retrograde trafficking of aberrantly localized phospholipids from the outer to the inner leaflet; when this system is inactivated or transcriptionally silenced, phospholipids accumulate in the outer leaflet, driving outward membrane curvature and vesicle release (Roier et al., 2016). This mechanism is conserved across phylogenetically distant Gram-negative species, including *Haemophilus influenzae, Vibrio cholerae*, and *Chromobacterium violaceum*, and vesicles produced by VacJ/Yrb-deficient strains contain approximately twice the phospholipid content of wild-type OMVs (Roier et al., 2016), illustrating how a single lipid-trafficking defect can quantitatively reshape vesicle membrane composition.

Protein incorporation into BEVs is similarly governed by identifiable sorting signals rather than passive co-sedimentation with the membrane. Outer membrane lipoproteins are covalently anchored to the underlying peptidoglycan via Braun's lipoprotein (Lpp); the fraction of Lpp that remains non-covalently associated, together with other loosely tethered periplasmic and outer membrane constituents, is preferentially released into nascent vesicles, whereas proteins with stable peptidoglycan-binding domains are retained in the cell envelope (Orench-Rivera and Kuehn, 2021). This principle was directly tested using the porin OmpA, whose C-terminal domain binds peptidoglycan: cells subjected to oxidative stress showed a marked, transcription-independent shift toward lipoprotein export and integral outer-membrane-protein retention, and this partitioning could be predicted from sequence-level features such as the presence of oxidizable residues (Orench-Rivera and Kuehn, 2021). Consistent with this cell-wall-anchoring model, the loading of the well-characterized porins OmpA and OmpC into *E. coli* OMVs is not fixed: comparative proteomic analyses of a hypervesiculating tolA mutant vs. wild-type *E. coli* found OmpA enrichment ratios in OMVs of 1.27-fold (ultracentrifugation) and 1.85-fold (ultrafiltration) relative to wild-type, with the magnitude of enrichment depending on both the genetic background and the isolation method used (Reimer et al., 2021). These findings indicate that even well-studied structural porins cannot be assumed to be uniformly represented across BEV preparations, a point directly relevant to their use as generic vesicle markers.

Beyond lipid trafficking and protein anchoring, post-translational and post-synthetic chemical modifications of BEV cargo constitute a third, functionally consequential axis of heterogeneity. The acylation state of lipid A, the membrane anchor and principal endotoxic moiety of lipopolysaccharide, is a particularly well-characterized example. Enzymes such as the 3-O-deacylase PagL and the palmitoyltransferase PagP remodel lipid A acyl chains in a PhoP/PhoQ-regulated manner, and deacylated or palmitoylated lipid A species activate human and murine TLR4 10- to 100-fold less potently than unmodified, fully acylated lipid A (Kawasaki et al., 2004). The magnitude of this effect can be substantially larger in engineered systems: outer membrane particles (GMMA) from *Shigella sonnei* and *S. flexneri* strains lacking the late acyltransferase genes msbB or htrB, which produce under-acylated lipid A, showed 600- to 60,000-fold reductions in TLR4 activation and 300- to 800-fold reductions in inflammatory cytokine induction compared with particles bearing wild-type, fully acylated lipid A (Rossi et al., 2014). Because acylation state is itself influenced by growth phase, temperature, and host-associated signals, BEVs isolated from the same strain under different conditions can differ by several orders of magnitude in their capacity to activate innate immune signaling, independent of any change in overall vesicle yield or protein content.

This compositional heterogeneity extends beyond immune activation to the routes by which BEVs are taken up by host cells and, by extension, to their distribution within tissues. BEV diameters span a wide range (approximately 20–500 nm), and this size heterogeneity influences the predominant entry pathway: vesicles that overlap in size with clathrin-coated pits (up to approximately 200 nm) are preferentially internalized via clathrin-mediated endocytosis, whereas larger or smaller vesicles may instead engage macropinocytosis, caveolin-dependent uptake, or direct membrane fusion (O'Donoghue and Krachler, 2016). Independently of size, the structure of surface-exposed lipopolysaccharide (LPS) also measurably alters uptake kinetics, with modified LPS glycoforms producing significantly different host-cell entry rates than the parental structure in time-resolved uptake assays (O'Donoghue et al., 2017). Collectively, these observations indicate that lipid composition, protein sorting, and cargo modification do not merely generate compositional “noise” between BEV preparations; they quantitatively determine the immunogenicity, uptake route, and likely biodistribution of the resulting vesicles, and therefore represent a central, mechanistically grounded challenge for the functional comparability, standardization, and clinical translation of BEV-based research and products.

### 2.3 Environmental influence and adaptive significance

Environmental conditions strongly influence both BEV production and cargo composition. Envelope stress, nutrient limitation, oxidative stress, and exposure to antimicrobial compounds can all alter vesiculation dynamics (Schwechheimer and Kuehn, 2015; Toyofuku et al., 2023; Kulp and Kuehn, 2010). Under these conditions, BEVs may contribute to bacterial adaptation through several mechanisms, including:

- Export of misfolded or toxic periplasmic material,
- Sequestration of harmful agents such as antibiotics or bacteriophages,
- Redistribution of enzymes and metabolites that support survival under stress conditions.

Changes in growth conditions can substantially modify vesicle composition and biological activity. As a result, BEVs isolated under different experimental settings may not be directly comparable, which remains an important issue for reproducibility and data interpretation (Schwechheimer and Kuehn, 2015; Toyofuku et al., 2023).

### 2.4 BEVs as protected delivery platforms

BEVs can function as carriers for molecules that are unstable or poorly diffusible in extracellular environments. Within microbial communities, vesicles have been implicated in the transport of quorum-sensing (QS) molecules, including autoinducers. Encapsulation may reduce signal dilution and protect signaling molecules from enzymatic degradation, particularly in spatially structured microbial environments (Xu et al., 2026). This could extend the stability and effective range of microbial communication.

### 2.5 Analytical challenges and future directions

Despite recent methodological advances, characterization of vesicle heterogeneity remains difficult. Common isolation procedures frequently yield mixed preparations containing BEVs together with protein aggregates, flagellar fragments, and other non-vesicular material (Schwechheimer and Kuehn, 2015; Toyofuku et al., 2023). Consequently, some reported cargo profiles may partly reflect technical contamination rather than biologically distinct vesicle populations.

Further progress in the field will likely require improved single-vesicle analytical methods, high-resolution imaging approaches, and integrated multi-omics strategies capable of distinguishing vesicle subpopulations at higher resolution. Better discrimination between vesicle types will be important for linking specific BEV populations to defined biological functions.

Overall, BEV biogenesis is shaped by both bacterial physiology and environmental conditions. Selective cargo incorporation appears to play an important role in microbial communication, host interaction, and pathogenesis, while also influencing the potential therapeutic applications of BEVs.

## 3 Role of BEVs in microbial communication

Bacterial extracellular vesicles (BEVs) contribute to microbial communication by enabling the transport of signaling molecules beyond direct cell-to-cell contact and simple diffusion processes. Through vesicle-mediated transport, bacteria can package and protect signaling cargo and deliver it to nearby microbial populations or host-associated environments. This mode of communication is particularly relevant in dense and spatially structured microbial communities, where diffusion alone may be insufficient for efficient signal transmission (Figure 3).

**Figure 3:**
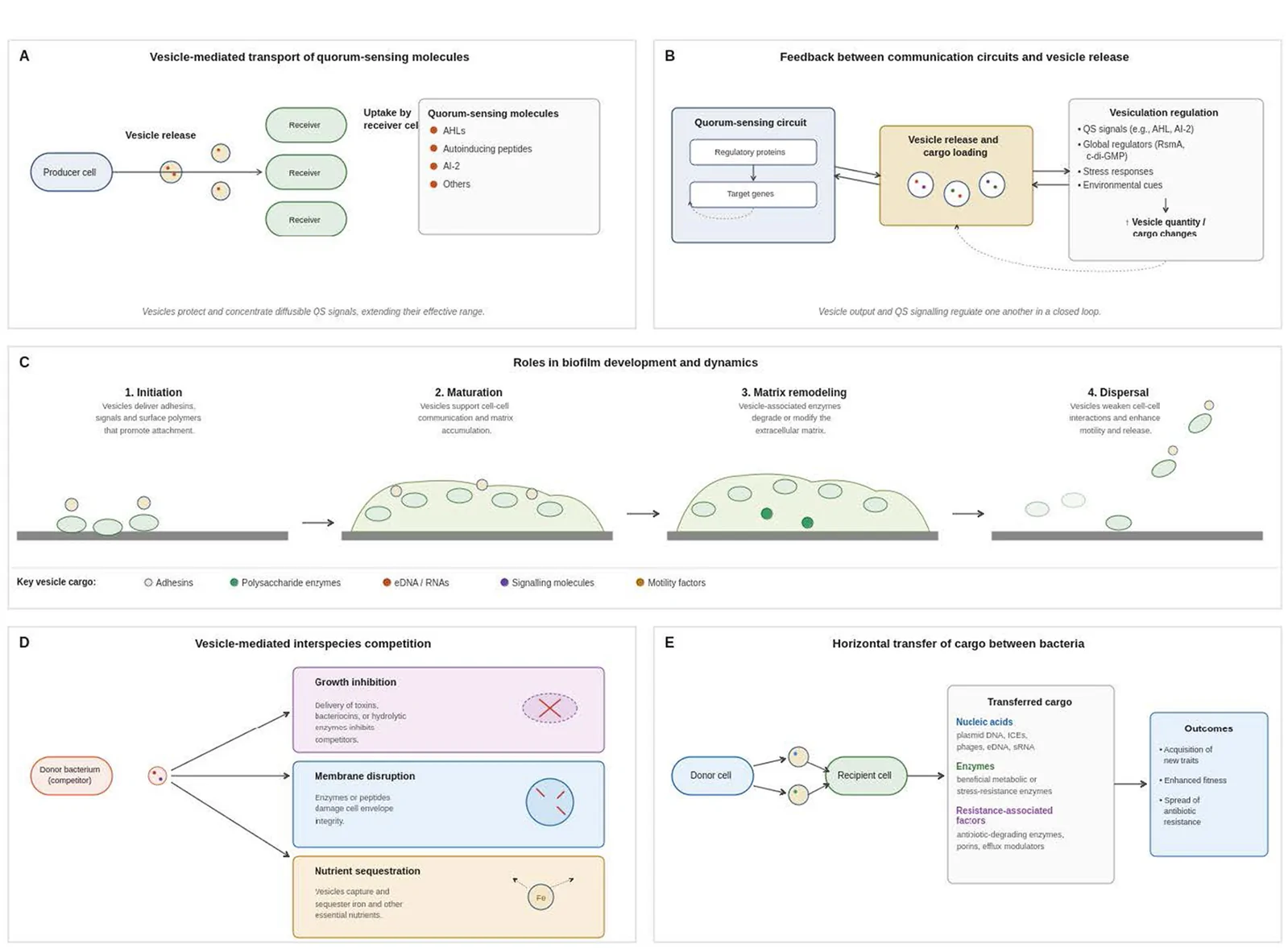
Roles of bacterial extracellular vesicles in microbial communication. Schematic representation of bacterial extracellular vesicle (BEV)-mediated processes in microbial communities, including quorum sensing, regulatory feedback loops, biofilm development, interspecies interactions, and horizontal transfer of biological cargo (Mashburn and Whiteley, 2005; Tsering et al., 2024; Nagasawa et al., 2025; Jeong et al., 2024; Tsai et al., 2025; Xiu et al., 2025; Kawagishi et al., 2025; Wen and Herman, 2024). **(A)** Vesicle-mediated transport of quorum-sensing molecules. **(B)** Feedback between communication circuits and vesicle release. **(C)** Roles of BEVs in biofilm development and dynamics (initiation, maturation, matrix remodeling, and dispersal). **(D)** Vesicle-mediated interspecies competition. **(E)** Horizontal transfer of biological cargo between bacteria.

### 3.1 Structured vectors for quorum sensing

One of the most studied communication-related functions of BEVs is their involvement in quorum sensing (QS). Conventional QS models rely largely on the extracellular diffusion of autoinducers, but signal stability can be limited in fluctuating or heterogeneous environments due to dilution and enzymatic degradation.

Several studies indicate that BEVs can encapsulate QS molecules within the vesicle lumen and transport them to recipient cells (Mashburn and Whiteley, 2005). Vesicle-associated packaging may help preserve signal integrity and allow localized delivery of signaling molecules at higher concentrations than would occur through free diffusion alone. This mechanism could support more stable and spatially restricted communication within microbial communities.

### 3.2 Feedback architectures: communication-driven vesiculation

The relationship between bacterial extracellular vesicles (BEVs) and bacterial communication is bidirectional. Signaling pathways not only exploit vesicles as effector vehicles but can also regulate their biogenesis. In *Streptococcus mutans*, for instance, cytoplasmic membrane vesicle (CMV) formation is linked to the Com signaling system, a peptide-mediated cell–cell communication pathway. In this system, vesicle biogenesis depends on autolysin activity that is itself regulated by Com signaling components (Nagasawa et al., 2025).

This coupling integrates vesicle production into broader bacterial regulatory circuits, establishing a feedback architecture in which vesiculation functions both as a downstream output of signaling and as a potential carrier of signaling-associated molecules (Mashburn and Whiteley, 2005; Nagasawa et al., 2025).

### 3.3 Dynamic regulation of biofilm architecture

BEVs play multifunctional roles in biofilm development, influencing adhesion, matrix organization, maturation, and dispersal (Jeong et al., 2024). Importantly, their effects are highly context-dependent, with evidence supporting both promotive and inhibitory outcomes.

- Biofilm inhibition: stress-induced vesiculation, such as imipenem-triggered outer membrane vesicles (OMVs) from *Elizabethkingia anophelis*, has been shown to impair biofilm formation and reshape microbial population dynamics in polymicrobial communities (Tsai et al., 2025).
- Biofilm promotion: conversely, vesicles from *Vibrio alginolyticus* contribute to biofilm development, where vesicle-associated small RNAs (sRNAs) are implicated in regulating extracellular matrix production and biofilm-associated phenotypes (Xiu et al., 2025).

### 3.4 Interspecies competition and pathogenicity modulation

Beyond intra-community regulation, BEVs also mediate interspecies interactions that influence microbial fitness and virulence. A recent study demonstrated that *Escherichia coli*-derived vesicles suppress the growth and virulence of Group A Streptococcus (GAS). This effect involves disruption of bacterial cell division, reprogramming of transcriptional activity, and downregulation of virulence-associated genes, ultimately resulting in reduced pathogenicity *in vivo* (Kawagishi et al., 2025).

These findings point to a role for BEVs in interspecies competition, with vesicle-mediated interactions capable of significantly modulating disease-relevant phenotypes.

### 3.5 Horizontal material transfer and antimicrobial resistance

BEVs are increasingly recognized as vehicles for horizontal transfer of biological cargo, including DNA, RNA, and functional proteins (Tsering et al., 2024; Wen and Herman, 2024). This mechanism is particularly relevant in the context of antimicrobial resistance (AMR), as vesicles can disseminate resistance determinants and enzymatic factors such as β-lactamases.

Within biofilms where high cell density and extracellular matrix structures enhance molecular retention BEV-mediated transfer may act in concert with classical horizontal gene transfer mechanisms such as conjugation and transformation. Although the quantitative contribution of BEVs to gene transfer remains under investigation, their role in maintaining genetic and functional plasticity under selective pressure is increasingly recognized (Wen and Herman, 2024; Chen et al., 2026).

### 3.6 Synthesis: toward a vesicle-centered view of microbial interaction

The evidence reviewed in this section supports a conceptual shift: BEVs are not simply byproducts of bacterial physiology but integral components of microbial interaction networks. By packaging signaling molecules, redistributing regulatory cargo, and facilitating genetic exchange, they enable bacterial populations to engage in spatially organized and functionally diverse modes of communication (Mashburn and Whiteley, 2005; Nagasawa et al., 2025; Kawagishi et al., 2025).

This vesicle-centered framework positions BEVs as central elements in microbial ecology and as mechanistic links connecting bacterial physiology, community behavior, and pathogenesis.

## 4 Role of BEVs in host interactions and immunomodulation

A central paradigm in contemporary microbiology is that bacteria can exert profound effects on host physiology without direct cellular contact or invasion. In this framework, bacterial extracellular vesicles (BEVs) emerge as key mediators of “action-at-a-distance” communication. These nanoscale, lipid bilayer–enclosed structures traverse mucosal barriers, interact with epithelial and immune interfaces, and deliver bioactive cargo in a protected and spatially regulated manner. Accumulating evidence positions BEVs as critical contributors to microbiota–host communication, shaping epithelial integrity, innate immune maturation, and inter-organ signaling across multiple physiological axes (Qian et al., 2025; Melo-Marques et al., 2024; Jeong et al., 2024; Ñahui Palomino et al., 2021).

### 4.1 The intestinal epithelium as a primary interface for vesicular signaling

The intestinal epithelium represents the principal interface for BEV–host interactions. At this site, BEVs exhibit marked context-dependent functional plasticity, with outcomes ranging from barrier reinforcement to epithelial dysfunction, depending on vesicle origin and molecular cargo (Melo-Marques et al., 2024; Nie et al., 2025; Han et al., 2024; Preet et al., 2025; Ñahui Palomino et al., 2021).

- Commensal-derived BEVs are generally associated with maintenance of epithelial homeostasis, including upregulation of tight junction proteins, attenuation of inflammatory injury, and promotion of mucosal repair mechanisms.
- Pathogen-derived BEVs, in contrast, frequently carry virulence-associated factors that disrupt epithelial integrity, enhance oxidative stress, increase paracellular permeability, and activate pro-inflammatory signaling cascades (Ñahui Palomino et al., 2021).

This functional dichotomy marks BEVs as key modulators of mucosal homeostasis and central determinants of dysbiosis-associated pathology.

### 4.2 Postbiotic potential: functional insights from probiotic-derived BEVs

Emerging evidence highlights the therapeutic relevance of BEVs derived from beneficial microbes, particularly probiotic taxa, which can recapitulate host-modulatory effects of their parental strains. These vesicles are increasingly conceptualized as “postbiotic” effector systems with intrinsic bioactivity.

A recent study demonstrated that BEVs from *Bifidobacterium longum* NSP001 ameliorated experimental colitis by enhancing epithelial barrier integrity and modulating metabolic pathways, including short-chain fatty acid–associated signaling. Notably, these effects persisted under germ-depleted conditions, supporting the notion that vesicles function as autonomous bioactive units (Nie et al., 2025).

Similarly, outer membrane vesicles from *Escherichia coli* Nissle 1917 enhance epithelial barrier function and attenuate inflammatory damage in colitis models, further reinforcing the translational relevance of probiotic-derived vesicles in intestinal inflammatory disorders (Han et al., 2024).

### 4.3 Modulation of innate and adaptive immunity

BEVs constitute highly immunostimulatory entities due to their enriched display of microbe-associated molecular patterns (MAMPs), including lipopolysaccharides, peptidoglycan fragments, and nucleic acids. This molecular architecture enables efficient engagement of pattern-recognition receptors (PRRs), particularly Toll-like receptors (TLRs), thereby triggering downstream inflammatory and regulatory signaling cascades (Qian et al., 2025; Melo-Marques et al., 2024; Marquez-Paradas et al., 2025).

Beyond innate immune activation, BEVs also contribute to adaptive immune modulation. Through delivery of antigenic and immunoregulatory cargo to antigen-presenting cells, they influence T-cell priming, polarization, and systemic immune calibration (Nie et al., 2025; Preet et al., 2025; Kozakiewicz et al., 2025). Depending on the immunological context, these processes may promote immune homeostasis or contribute to chronic inflammatory disease states.

### 4.4 Systemic dissemination and the organ-axis framework

A major conceptual advance in BEV biology is the recognition of their capacity for systemic dissemination beyond the intestinal lumen. Increasing evidence implicates BEVs in inter-organ communication along the gut–liver, gut–lung, gut–brain, and gut–heart axes (Zhou et al., 2025; Jang et al., 2015; Mottawea et al., 2025; Marquez-Paradas et al., 2025).

Among these, the gut–heart axis has received comparatively little attention despite mechanistic plausibility and a growing evidence base. Bacterial-derived vesicles are detectable in the bloodstream of healthy individuals, the majority of gut-microbiota origin, indicating that low-level BEV translocation across the intestinal epithelium is not restricted to conditions of overt barrier disruption; fluorescently labeled OMVs traverse the gastrointestinal mucosa and enter the circulation rapidly following oral administration even without induced intestinal injury (Lin et al., 2025; Jones et al., 2020). Once in the circulation, gut-derived BEVs have been implicated in promoting vascular inflammation and atherosclerotic plaque progression, with their surface and cargo components, including LPS and pro-inflammatory proteins, capable of activating endothelial and immune cells at distal vascular sites in a manner mechanistically analogous to the gut–liver and gut–brain signaling described above (Lin et al., 2025). Because cardiovascular disease shares many of the same dysbiosis- and barrier-disruption-associated risk factors already implicated in hepatic and neurological BEV signaling, the gut–heart axis represents a natural, mechanistically consistent extension of the organ-axis framework depicted in Figure 4, and is discussed here alongside the gut–liver, gut–lung, and gut–brain routes shown in that figure.

**Figure 4:**
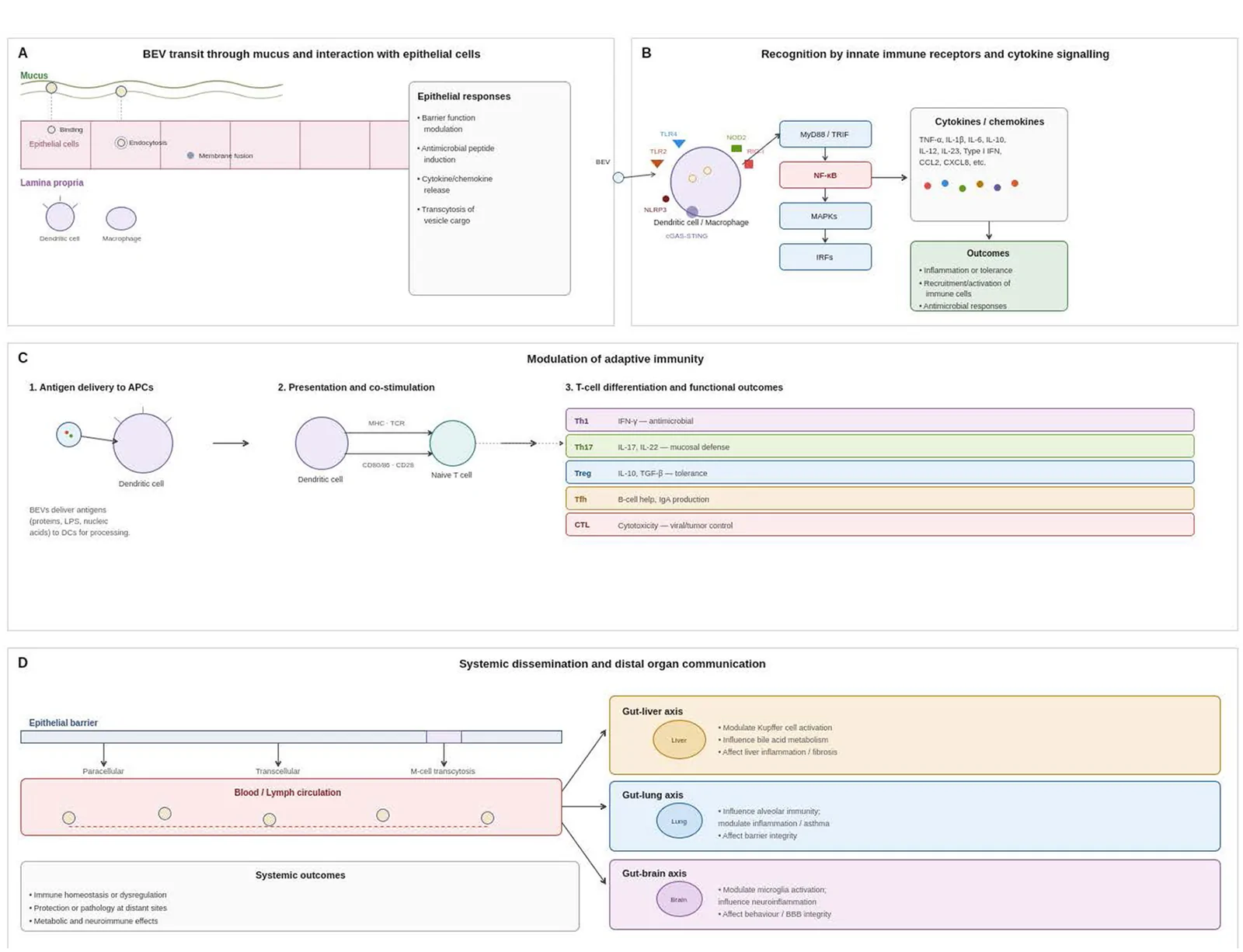
Bacterial extracellular vesicle (BEV)-mediated interactions with the host. Schematic representation of BEV interactions with the host, including epithelial barrier engagement, immune recognition and signaling, modulation of adaptive immunity, and systemic dissemination across host tissues [Qian et al., 2025; Melo-Marques et al., 2024; Lin et al., 2025; Jones et al., 2020; Nie et al., 2025; Han et al., 2024; Li Y. et al., 2026; Preet et al., 2025). **(A)** BEV transit through mucus and interaction with epithelial cells. **(B)** Recognition by innate immune receptors and downstream cytokine signaling. **(C)** Modulation of adaptive immunity, including antigen delivery, presentation, and T-cell differentiation. **(D)** Systemic dissemination and distal organ communication along the gut, liver, lung, and brain axes.

Notably, a study in *Nature Communications* demonstrated that gut-derived *Bifidobacterium* vesicles can modulate responses to anti–PD-1 immunotherapy in lung cancer models. Mechanistically, these vesicles were shown to regulate tumor-associated immune signaling, including TLR4–NF-κB–dependent pathways influencing immune checkpoint expression, thereby reshaping distal tumor microenvironments (Preet et al., 2025).

These findings support a model in which BEVs act as systemic signaling units, translating intestinal microbial activity into distal physiological and pathological outcomes that span metabolic regulation, hepatic function, and tumor immunity (Zhou et al., 2025; Jang et al., 2015; Mottawea et al., 2025).

### 4.5 Pathogenic vs. beneficial vesicular functions

Despite their homeostatic roles, BEVs can also contribute to disease pathogenesis depending on microbial origin and host context. Pathogen-derived vesicles often serve as concentrated delivery systems for toxins, immune-modulatory factors, and enzymatic activities that facilitate immune evasion and tissue damage.

Such vesicles can promote neutrophil recruitment, compromise epithelial barrier integrity, and amplify systemic inflammatory responses. This duality highlights the context-dependent nature of BEV biology, wherein a conserved structural platform can mediate either beneficial or pathogenic outcomes depending on ecological and molecular determinants (Qian et al., 2025; Melo-Marques et al., 2024; Ñahui Palomino et al., 2021; Marquez-Paradas et al., 2025).

### 4.6 Conceptual summary: BEVs as integrated functional units of host–microbiota biology

BEVs emerge from this section as compact, biologically active entities that integrate microbial physiology with host signaling networks. Through their capacity to modulate innate immune sensing, orchestrate adaptive immune responses, and mediate long-range inter-organ communication, they function as central intermediates in host–microbiota interactions.

This multifunctionality positions BEVs at the forefront of translational research, with growing relevance for the development of novel diagnostics, vaccine platforms, and immunomodulatory therapeutic strategies.

## 5 BEVs in pathogenesis, dysbiosis, and disease

The clinical relevance of bacterial extracellular vesicles (BEVs) derives from their role as concentrated carriers of virulence determinants, inflammatory mediators, and regulatory nucleic acids. In pathological contexts, BEVs act as potent effector systems that amplify dysbiosis, disrupt mucosal homeostasis, and remodel distal disease microenvironments. Crucially, their activity is not contingent on bacterial viability, enabling them to traverse epithelial barriers and disseminate systemically, thereby providing a mechanistic basis for chronic inflammatory phenotypes in the absence of overt infection or bacterial translocation (Table 2) (Qian et al., 2025; Ñahui Palomino et al., 2021; Marquez-Paradas et al., 2025; Sun et al., 2023).

**Table 2:** Beneficial vs. pathogenic effects of bacterial extracellular vesicles.

Feature	Beneficial BEVs	Pathogenic BEVs
Origin	Probiotic and commensal bacteria	Pathogenic and pathobiont bacteria
Barrier integrity	Reinforce tight junctions	Disrupt epithelial barriers
Immune response	Promote immune homeostasis	Trigger excessive inflammation
Major cargo	Immunomodulatory proteins, metabolites, regulatory RNAs	Toxins, virulence factors, and inflammatory molecules
Disease association	Colitis protection, immune regulation	IBD, CRC, liver disease, atherosclerosis
Therapeutic potential	Postbiotics, drug delivery, immunotherapy	Vaccine targets, biomarkers

### 5.1 Dysbiosis and the functional readout paradigm

Within dysbiotic microbial ecosystems, BEVs are increasingly conceptualized as *functional readouts* of microbial imbalance rather than passive by-products of bacterial growth. Compositional shifts in the microbiota are reflected in altered vesicle production and cargo composition, enabling the dissemination of disease-associated signals even when producer organisms remain confined to the intestinal lumen (Sun et al., 2023; Zubair et al., 2024).

This framework suggests that BEVs may exert systemically amplified effects relative to their microbial abundance, sustaining chronic immune activation even under conditions of reduced bacterial load (Ñahui Palomino et al., 2021; Marquez-Paradas et al., 2025; Sun et al., 2023).

### 5.2 Inflammatory bowel disease (IBD) and barrier disruption

In the inflamed intestinal environment, BEVs occupy a functionally ambivalent role. While commensal-derived vesicles support epithelial repair and immune resolution, vesicles originating from pathobionts exacerbate epithelial injury and perpetuate cytokine-driven inflammatory circuits (Qian et al., 2025; Melo-Marques et al., 2024; Ñahui Palomino et al., 2021; Marquez-Paradas et al., 2025).

This duality points to their central role in IBD pathophysiology. Accordingly, BEVs are increasingly recognized not only as mediators of disease exacerbation but also as mechanistic targets for therapeutic intervention, given their integration into epithelial barrier regulation and mucosal immune signaling networks (Pirolli et al., 2025b).

### 5.3 The oncogenic interface: colorectal cancer and beyond

BEVs have emerged as active contributors to the tumor microenvironment (TME), influencing oncogenesis through immune modulation, epithelial interaction, and microbial niche stabilization (Zheng et al., 2024; Chronopoulos and Kalluri, 2020; Luo et al., 2024).

- Microbial persistence: a pivotal 2024 study demonstrated that *Fusobacterium nucleatum*-derived vesicles are enriched in colorectal cancer (CRC) tissues, where they promote bacterial adhesion and facilitate niche persistence within the tumor microenvironment (Zheng et al., 2024).
- Mechanistic duality: the role of BEVs in cancer is increasingly understood as cargo-dependent rather than strictly taxon-dependent. While many vesicles promote tumor-supportive inflammation, a 2025 study reported that specific BEV-associated single-stranded RNAs (ssRNAs) may suppress CRC progression via Piezo1-dependent signaling pathways (Kondo et al., 2025).

These findings emphasize the necessity of moving beyond taxonomic attribution toward molecular cargo–based classification when evaluating BEV function in oncogenesis (Chronopoulos and Kalluri, 2020; Luo et al., 2024).

### 5.4 Systemic pathopathology: the gut–organ axes

BEV-mediated inter-organ communication represents a major axis through which microbial dysbiosis translates into systemic disease.

- Gut–liver axis: owing to portal circulation trafficking, BEVs represent a key conduit for microbial signals to the liver. A 2026 study identified that gut-derived *Klebsiella pneumoniae* vesicles induce immunosuppressive macrophage polarization in hepatic tissue, thereby facilitating bacterial translocation and potentially contributing to hepatocellular carcinoma (HCC) progression (Tsubaki et al., 2026).
- Cardiometabolic axis: in the gut–heart axis, pathogen-derived BEVs are implicated in endothelial dysfunction and foam cell formation within atherosclerotic lesions. This positions BEVs as underrecognized circulating effectors that contribute to sustained vascular inflammation and cardiometabolic disease progression (Lin et al., 2025; Wang et al., 2026).

### 5.5 BEVs as diagnostic biomarkers and liquid biopsies

Beyond their pathogenic roles, BEVs are emerging as stable, non-invasive biomarkers for disease detection. Their structural integrity and protected cargo enable preservation of source-specific molecular signatures in biological fluids, making them attractive candidates for microbial “liquid biopsy” approaches.

Elevated circulating BEV-associated microbial signatures have been reported in cancer cohorts relative to healthy controls, suggesting potential utility in early detection and disease stratification of dysbiosis-associated pathologies (Mehmood and Danaf, 2025).

### 5.6 Synthesis: from correlation to causality

BEVs occupy a mechanistically central position at the interface between microbial dysbiosis and host pathology, providing a plausible molecular link between intestinal microbial imbalance and systemic disease manifestations that include hepatic fibrosis, metabolic dysfunction, vascular inflammation, and oncogenesis.

A key unresolved challenge is the functional stratification of BEV populations into *driver* vesicles that actively propagate disease mechanisms and *passenger* vesicles that reflect underlying microbial states. Resolving this distinction will be critical for translating BEV biology into clinically actionable diagnostics and therapeutic strategies (Qian et al., 2025; Zhou et al., 2025; Jang et al., 2015; Sun et al., 2023; Chronopoulos and Kalluri, 2020; Wang et al., 2026; Mehmood and Danaf, 2025).

## 6 Beneficial and probiotic-derived BEVs: emergence of the postbiotic paradigm

Historically, bacterial extracellular vesicles (BEVs) were primarily interpreted through their roles in virulence and host injury. However, this view has been substantially revised by recent evidence demonstrating that vesicles derived from commensal and probiotic bacteria are active mediators of host–microbiota mutualism rather than inert by-products of microbial physiology. These vesicles contribute to epithelial integrity, immune maturation, and resilience to inflammatory stress, thereby occupying a central position within beneficial host–microbiota interactions (Melo-Marques et al., 2024; Zhang et al., 2025; Nie et al., 2025; Han et al., 2024; Marquez-Paradas et al., 2025; Kozakiewicz et al., 2025).

### 6.1 Restoration of mucosal homeostasis and barrier integrity

A recurring finding across recent studies is the capacity of beneficial BEVs to reinforce intestinal barrier function, a role traditionally attributed to live probiotic organisms. Probiotic-derived vesicles enhance epithelial integrity through upregulation of tight-junction proteins and suppression of pro-inflammatory signaling pathways, thereby promoting restoration of mucosal homeostasis under inflammatory conditions.

Importantly, this activity indicates that BEVs can reproduce key probiotic effects independently of bacterial viability or colonization. This is particularly relevant in clinical settings where administration of live microorganisms is contraindicated, such as in immunocompromised hosts or conditions associated with increased risk of microbial translocation (Zhang et al., 2025; Nie et al., 2025; Han et al., 2024; Kozakiewicz et al., 2025).

### 6.2 Mechanistic insights from key commensal taxa

Recent experimental work has begun to delineate the mechanistic diversity of commensal-derived BEVs:

- *Bifidobacterium longum* NSP001: vesicles derived from this strain ameliorate experimental colitis through modulation of short-chain fatty acid (SCFA)–associated metabolic pathways and regulation of immune cell differentiation. Their efficacy under microbiota-depleted conditions further indicates that their activity is not solely ecosystem-dependent but involves direct vesicle–host interactions (Nie et al., 2025; Preet et al., 2025).
- *Escherichia coli* Nissle 1917: outer membrane vesicles (OMVs) from this well-characterized probiotic strain exhibit robust anti-inflammatory activity. Their therapeutic potential is further enhanced through incorporation into engineered delivery systems, supporting their use as modular postbiotic platforms (Han et al., 2024).
- Emerging beneficial taxa: broader analyses identify vesicles from *Lactobacillus* spp., *Akkermansia muciniphila*, and *Faecalibacterium prausnitzii* as important regulators of mucosal immune homeostasis. These vesicles carry bioactive proteins, lipids, and metabolite-associated cargo that collectively shape epithelial and immune functions (Zhang et al., 2025; Hu et al., 2021; Kozakiewicz et al., 2025).

### 6.3 Precision immunoregulation

In contrast to broad immunosuppressive agents, beneficial BEVs appear to mediate *context-dependent immune calibration* rather than global immune suppression. They interact with antigen-presenting cells (APCs) and lymphocyte subsets to promote tolerogenic signaling pathways and support regulatory T-cell (Treg) differentiation.

This selective immunomodulatory behavior supports a model in which BEVs sustain immune equilibrium by reinforcing physiological tolerance mechanisms rather than merely attenuating inflammatory responses. Such properties are increasingly recognized as defining features of functionally relevant postbiotic candidates (Nie et al., 2025; Marquez-Paradas et al., 2025; Kozakiewicz et al., 2025).

### 6.4 BEVs as postbiotic platforms: translational advantages and constraints

The conceptual transition from live probiotics to BEV-based postbiotics offers several translational advantages. These include improved safety due to the absence of replicative organisms, reduced risk of horizontal gene transfer, and greater potential for standardization of dosage and molecular composition. In addition, their inherent stability facilitates incorporation into engineered delivery systems and pharmaceutical formulations.

However, BEV functionality is highly sensitive to bacterial growth conditions, strain-specific physiology, and isolation methodologies. Accordingly, their classification as “beneficial” should be considered context-dependent rather than intrinsic. Robust validation of strain specificity, cargo consistency, and functional reproducibility remains essential for clinical translation (De Langhe et al., 2024; Toyofuku et al., 2023; Kozakiewicz et al., 2025).

### 6.5 Safety considerations for postbiotic BEV applications

Beyond their beneficial and immunomodulatory functions, BEVs derived from both pathogenic and commensal bacteria carry molecular cargo capable of eliciting adverse host responses, and these safety considerations warrant explicit discussion before BEVs can be advanced as postbiotic therapeutics. In Gram-positive bacteria, lipoteichoic acid (LTA) is anchored in the cytoplasmic membrane and, together with other membrane lipids, is packaged into cytoplasmic membrane vesicles during biogenesis (Section 2.1). LTA is recognized by Toll-like receptor 2 (TLR2) and, together with peptidoglycan fragments, is regarded as a principal virulence factor underlying inflammatory pathology in Gram-positive infection, with reported associations ranging from localized skin inflammation to septic shock (Kang et al., 2016). Because LTA structure and acylation vary considerably between and within Gram-positive genera, and because commensal and probiotic strains are not inherently exempt from producing immunostimulatory LTA variants, vesicles from beneficial Gram-positive taxa cannot be assumed *a priori* to be immunologically inert; LTA content and TLR2 agonism should therefore be included as a standard component of BEV safety characterization, analogous to the lipid A/TLR4 assessment already emphasized for Gram-negative OMVs (Section 2.2).

A second safety consideration concerns the capacity of BEVs to package and horizontally transfer mobile genetic elements, including plasmids and phage-derived DNA, independently of direct cell-to-cell contact. Outer membrane vesicles from *Klebsiella pneumoniae* protect plasmid DNA from extracellular nuclease degradation and transfer plasmid-encoded antibiotic-resistance genes to recipient *Escherichia coli, Salmonella enterica, Pseudomonas aeruginosa*, and *Burkholderia cepacia* at efficiencies that scale with plasmid copy number, with transformation efficiency differing by 3.6-fold between a high- and a low-copy-number plasmid (Dell'Annunziata et al., 2021). Similarly, *Pseudomonas aeruginosa* OMVs transfer plasmid-encoded resistance genes to recipient bacteria more efficiently than free plasmid DNA alone, and vesicles derived from biofilm cultures are more efficient vectors for transformation than those from planktonic cultures despite being smaller in size (Johnston et al., 2023). These findings indicate that BEV-based products, including those intended for oral or topical postbiotic use, could in principle disseminate resistance or virulence determinants within recipient microbial communities; this risk is generally considered lower than that posed by replication-competent live biotherapeutics, since BEVs themselves cannot replicate or persist indefinitely, but it is not zero and merits explicit risk assessment rather than being assumed away.

A third consideration relates to the systemic fate of BEVs following oral or intestinal exposure, which is directly relevant to their proposed use as orally delivered postbiotics. Fluorescently labeled *Bacteroides thetaiotaomicron* outer membrane vesicles administered orally to healthy mice were found to translocate across the intestinal epithelium and accumulate predominantly in the gastrointestinal tract and liver, indicating that an intact mucosal barrier does not preclude systemic BEV translocation (Jones et al., 2020). Consistent with this, intraperitoneal administration of *Escherichia coli* OMVs in mice reproduces a sepsis-like systemic inflammatory syndrome with elevated TNF-α and IL-6, hypothermia, and lethality at sufficient doses (Park et al., 2010), while gut microbiota-derived vesicles administered orally to diabetic rats and to renal epithelial cells *in vitro* have been linked to chronic tubulointerstitial inflammation and kidney injury distant from the site of administration (Chen et al., 2023). Although these effects have been documented predominantly with vesicles from pathogenic or dysbiosis-associated taxa rather than with well-characterized probiotic strains, they establish that BEVs are not confined to the gut lumen after oral delivery, and that both dose and bacterial source should be considered when evaluating the safety of BEV-based oral therapeutics, rather than extrapolating safety directly from the innocuity of the parent live organism.

These three considerations—immunostimulatory lipid content, vector potential for mobile genetic elements, and systemic biodistribution following oral exposure—together indicate that the translational advantages attributed to BEV-based postbiotics in Section 6.4 should be understood as relative rather than absolute. A defined safety characterization panel, analogous to the compositional standards discussed in Section 7, will be necessary before BEV-based products can be advanced with confidence into clinical use.

### 6.6 From postbiotic promise to clinical reality

Beneficial and probiotic-derived BEVs represent a mechanistic bridge between microbiome ecology and therapeutic innovation. By mediating host protection through discrete nanoscale signaling units, they provide a refined conceptual framework for understanding how commensal bacteria exert systemic physiological effects.

Moving toward clinical application, key challenges include resolving vesicle heterogeneity, standardizing production and purification workflows, and establishing robust functional benchmarks. Addressing these limitations will be critical for realizing the full translational potential of BEVs as next-generation postbiotic therapeutics and precision immunomodulatory platforms (Zhang et al., 2025; Nie et al., 2025; Han et al., 2024; Marquez-Paradas et al., 2025; Kozakiewicz et al., 2025).

## 7 Methodological challenges: isolation, purification, and the quest for standardization

The rapidly expanding interest in bacterial extracellular vesicles (BEVs) is accompanied by persistent methodological limitations that directly affect reproducibility, interpretability, and translational feasibility (De Langhe et al., 2024; Toyofuku et al., 2023; Wang et al., 2026). These constraints are not merely technical in nature; they actively shape the biological conclusions derived from vesicle-based studies. Given their nanoscale dimensions, structural heterogeneity, and diverse biogenetic origins, even minor variations in experimental workflows can substantially alter vesicle yield, cargo composition, and downstream functional readouts (De Langhe et al., 2024; Nagasawa et al., 2025).

### 7.1 Harmonization of isolation and purification strategies

A central unresolved issue in BEV research is the absence of a universally accepted gold-standard isolation protocol. Current workflows typically combine differential ultracentrifugation (UC), density gradient centrifugation, size-exclusion chromatography (SEC), and affinity-based enrichment approaches (De Langhe et al., 2024; Welsh et al., 2024; Wang et al., 2026).

Although UC remains widely used due to accessibility and scalability, it is prone to co-isolation of non-vesicular contaminants, including protein aggregates, flagellar fragments, bacteriophages, and membrane debris (De Langhe et al., 2024; Welsh et al., 2024). By contrast, SEC and density gradient methods generally improve purity but introduce trade-offs in yield and may impose size- or density-dependent selection biases that distort the recovered vesicle population (De Langhe et al., 2024; Wang et al., 2026).

As a result, BEV preparations generated across laboratories or even across experimental batches within the same laboratory often lack direct comparability, representing a major obstacle for meta-analytic integration and cross-study synthesis.

Direct comparisons among the three most widely used isolation strategies highlight consistent, method-specific trade-offs, although most head-to-head benchmarking to date has been performed with eukaryotic small extracellular vesicles rather than BEVs specifically, and the relative rankings should be interpreted with this caveat in mind. In plasma-derived small extracellular vesicles, ultracentrifugation (UC) yields fewer particles than size-exclusion chromatography (SEC) but achieves markedly higher overall purity; SEC preparations show higher particle counts, protein content, and particle-to-protein ratios, but these apparent gains are partly offset by co-elution of apolipoprotein-positive lipoproteins and other non-vesicular protein, illustrating that particle-to-protein ratio alone can be a misleading purity proxy when lipoprotein contamination is present (Takov et al., 2019). Tangential flow filtration (TFF) offers a further alternative: in head-to-head comparisons with UC (both followed by a common SEC purification step) across several human and murine cell lines, TFF recovered significantly higher vesicle yields than UC at comparable size distributions while requiring less specialized equipment, making it an attractive option for scaling isolation to larger sample volumes (Visan et al., 2022). Although these comparisons were not performed directly on BEVs, the same underlying trade-off between yield, purity, and scalability is expected to apply and has been explicitly addressed in fecal BEV workflows specifically: for fecal and other intestinal BEV preparations, orthogonal workflows that combine sequential filtration, SEC, and density gradient ultracentrifugation are increasingly used to separate BEVs from bacterial cells, flagella, and cell debris; a recently optimized bottom-up density-gradient protocol achieved a working fecal BEV concentration of approximately 10^8^ particles per milligram of starting material within a 7-h centrifugation step, compared with up to 18 h required by earlier protocols, illustrating that meaningful gains in both throughput and reproducibility remain achievable without sacrificing recovery or purity (Xue et al., 2023). No single method is universally superior across all three axes of recovery efficiency, purity, and preservation of biological activity; the appropriate choice instead depends on whether a given study prioritizes maximal yield (favoring TFF or UC), maximal purity for compositional profiling (favoring SEC or density gradients), or a balance of both for functional assays, and this trade-off should be stated explicitly when BEV preparations are reported.

### 7.2 Cargo authenticity vs. extracellular contamination

The biological interpretation of BEV cargo critically depends on the stringency of purification workflows. Omics-based studies frequently assign proteins, nucleic acids, and metabolites as vesicle-associated components; however, a substantial fraction of these molecules may originate from co-purified extracellular material rather than true intravesicular or membrane-associated cargo (De Langhe et al., 2024; Welsh et al., 2024; Wang et al., 2026).

This distinction is particularly consequential when inferring selective cargo loading mechanisms or proposing diagnostic biomarkers. In the absence of rigorous enzymatic treatments (e.g., protease or nuclease digestion of extravesicular material), there is a significant risk of misattributing soluble or environmental molecules as vesicle-encapsulated signals (Welsh et al., 2024; Wang et al., 2026). This remains one of the primary sources of interpretational uncertainty in BEV functional studies.

A related and particularly acute problem in intestinal and fecal BEV research is distinguishing genuine bacterial vesicles from other extracellular particles that overlap substantially in size and buoyant density, most notably bacteriophages and host-derived extracellular vesicles. Because most bacteriophage virions and BEVs both fall within the approximately 20–300 nm size range and can co-sediment during ultracentrifugation or co-elute during SEC, purity based on particle counts or size distribution alone is insufficient to confirm vesicle identity. The problem is compounded by direct physical association between the two entities: outer membrane vesicles have been shown to remain stably attached to filamentous phage particles through multiple purification steps, and this vesicle-phage complex has measurable, distinct immunomodulatory activity compared with either component alone, meaning that incomplete separation does not merely dilute a BEV preparation but can actively confound its functional interpretation (Pennetzdorfer et al., 2023). In fecal samples, this challenge is compounded further by the presence of host-derived extracellular vesicles shed from the intestinal epithelium and immune cells, which share the same size range as BEVs and cannot be excluded by filtration or density gradients alone. Reliable discrimination therefore requires orthogonal, composition-based confirmation rather than physical separation alone: nuclease and protease pretreatment to remove non-encapsulated nucleic acids and surface protein contaminants, bacterial-specific molecular markers (e.g., LPS, peptidoglycan-associated proteins, or 16S rRNA gene content for taxonomic assignment) to confirm bacterial origin, and, where phage contamination is a specific concern, DNase-protection assays or capsid-protein immunolabeling to exclude encapsidated phage genomes from vesicle-associated nucleic acid pools.

### 7.3 Resolution of vesicle heterogeneity

BEVs are frequently treated as a homogeneous entity, despite representing a heterogeneous mixture of vesicle subtypes derived from distinct biogenetic pathways. In Gram-negative bacteria, canonical outer membrane vesicles (OMVs) coexist with outer–inner membrane vesicles (OIMVs), the latter often associated with cell lysis and enriched in cytoplasmic constituents such as DNA, RNA, and metabolic enzymes (De Langhe et al., 2024; Schwechheimer and Kuehn, 2015; Wang et al., 2026).

In Gram-positive bacteria, vesicle heterogeneity is even more pronounced, reflecting species-specific differences in cell wall architecture, enzymatic remodeling, and secretion dynamics (Jones et al., 2020; Wang et al., 2026). Failure to resolve these subpopulations can obscure functionally dominant yet numerically minor vesicle subsets, leading to oversimplified or potentially misleading biological interpretations.

### 7.4 Environmental and culture-dependent variability

BEV production is highly sensitive to environmental parameters, including nutrient availability, oxygen tension, pH, and exposure to antimicrobial agents (De Langhe et al., 2024; Toyofuku et al., 2023; Mehmood and Danaf, 2025). These conditions influence not only vesicle abundance but also cargo composition, surface properties, and biophysical characteristics.

This environmental plasticity introduces a critical translational limitation: vesicles generated under *in vitro* conditions may not faithfully recapitulate those produced *in vivo* within polymicrobial or host-associated ecosystems. This discrepancy is particularly relevant for clinical translation, including vaccine development and postbiotic applications, where reproducibility and physiological relevance are essential regulatory requirements (Welsh et al., 2024; Wang et al., 2026).

### 7.5 Integrated characterization and reporting standards

Conventional characterization techniques such as nanoparticle tracking analysis (NTA) and electron microscopy (EM) are indispensable but insufficient when applied in isolation. High-confidence BEV studies require integrated, multi-parameter characterization frameworks incorporating particle concentration, morphological validation, purity metrics (e.g., protein-to-particle ratios), and functional validation assays (De Langhe et al., 2024; Welsh et al., 2024; Wang et al., 2026).

In addition, bacterial systems require specific controls to account for confounders such as spontaneous cell lysis and bacteriophage contamination variables that are comparatively less prominent in eukaryotic extracellular vesicle research but critically important in microbial systems (De Langhe et al., 2024; Wang et al., 2026).

### 7.6 Toward a minimal characterization and reporting panel

The considerations outlined above point toward a practical, adoptable minimum characterization panel rather than an aspirational ideal. Building on the precedent set by the Minimal Information for Studies of Extracellular Vesicles (MISEV) guidelines developed for eukaryotic EVs (Welsh et al., 2024), a recent call for consensus in bacterial EV research has proposed that comparable minimal reporting standards are both feasible and overdue for BEVs specifically (Choi and Lee, 2025). At minimum, such a panel should report: (i) particle concentration and size distribution by at least one orthogonal sizing method (e.g., nanoparticle tracking analysis paired with electron microscopy); (ii) a purity metric such as the particle-to-protein ratio, alongside explicit testing for non-vesicular contaminants relevant to the sample matrix (soluble aggregates, phage particles, host-derived vesicles); (iii) confirmation of bacterial membrane origin using at least one compositional marker (e.g., LPS or lipoteichoic acid content, or a defined outer-membrane or cytoplasmic-membrane protein marker); and (iv) full disclosure of growth conditions, growth phase at harvest, and the specific isolation workflow used, given the marked sensitivity of BEV yield and cargo composition to these variables (Sections 2.3 and 7.4).

Reference materials and batch-to-batch quality control represent a further, largely unmet need. Unlike eukaryotic EV research, which increasingly relies on well-characterized reference vesicle preparations and spike-in standards, bacterial EV studies rarely benchmark new preparations against a shared reference material, making it difficult to determine whether observed differences between studies reflect genuine biology or batch-specific technical variation. A practical interim solution is for individual laboratories to establish and characterize their own internal reference lot for each strain and condition used routinely, against which subsequent preparations are compared using the panel above, together with a pre-specified acceptance range (e.g., particle-to-protein ratio within a defined fold-range of the reference lot) before a batch is used in downstream functional or translational experiments. Wider adoption of shared, community-deposited reference BEV materials, analogous to those maintained for eukaryotic EVs, would substantially strengthen cross-study comparability as the field matures.

### 7.7 Standardization as a foundational requirement for the field

There is growing consensus that methodological standardization is not merely a technical refinement but a foundational requirement for the maturation of BEV research. This includes harmonized terminology, transparent reporting of isolation workflows, and implementation of orthogonal validation strategies to distinguish bona fide vesicles from extracellular artifacts (De Langhe et al., 2024; Welsh et al., 2024; Wang et al., 2026).

Ultimately, progress in the field will depend on achieving reproducible vesicle isolation and robust cross-study comparability. Without such standardization, mechanistic insights and translational applications will remain fragmented and difficult to validate at scale (De Langhe et al., 2024; Welsh et al., 2024; Pawar et al., 2025; Fujikawa et al., 2025).

## 8 Therapeutic, diagnostic, and vaccine applications of BEVs

The translational potential of bacterial extracellular vesicles (BEVs) lies in the convergence of intrinsic biological functionality with the structural versatility of engineered nanocarriers. As nanoscale, lipid-bilayer–enclosed systems capable of encapsulating diverse molecular cargo, BEVs are uniquely positioned to interface with both microbial ecosystems and host biological systems (De Langhe et al., 2024; Qian et al., 2025; Toyofuku et al., 2023; Zou et al., 2025). The current translational landscape can be broadly categorized into three principal domains: non-invasive diagnostics, vaccine development, and engineered therapeutic delivery. While outer membrane vesicle (OMV)-based vaccines represent the most clinically advanced application, the use of BEVs as precision diagnostic tools and programmable therapeutic platforms is rapidly emerging as a central direction in microbiome-based nanomedicine (De Langhe et al., 2024; Zou et al., 2025; Jeong et al., 2024; Asano-Inami et al., 2025).

### 8.1 BEVs as diagnostic and biomarker platforms: toward a microbial liquid biopsy

BEVs offer a distinct diagnostic advantage due to their enhanced stability in complex biological fluids compared to free nucleic acids or soluble microbial proteins. Their lipid bilayer confers protection against enzymatic degradation, preserving encapsulated molecular cargo that reflects the physiological and functional state of the originating microorganism (Qian et al., 2025; Marquez-Paradas et al., 2025; Mehmood and Danaf, 2025).

In pathological contexts such as cancer and chronic inflammation, BEV-associated cargo functions as a dynamic readout of microbial adaptation to host microenvironments. Recent studies have identified disease-associated BEV-derived DNA, protein, and metabolite signatures in gastric and colorectal cancer cohorts, supporting a shift from taxonomic profiling toward mechanism-informed diagnostic frameworks (Mehmood and Danaf, 2025; Zhang et al., 2026; Bhanu et al., 2025).

This represents a conceptual advance over conventional approaches such as 16S rRNA sequencing, as BEV-based profiling captures active microbial output rather than static community composition. However, clinical translation remains contingent on overcoming methodological variability, particularly in isolation workflows and downstream analytical standardization, which currently limits cross-study comparability and diagnostic robustness (De Langhe et al., 2024; Welsh et al., 2024; Wang et al., 2026).

A recent proof-of-concept study illustrates how such BEV-derived signals can be translated into a quantitative clinical index. Using 16S rRNA metagenomic analysis of serum extracellular vesicles from patients with renal cell carcinoma, three bacterial DNA taxa (Bacteroidia, TM7-1, and Sphingomonadales) were found to be differentially abundant relative to healthy donors; combined into a composite index, they distinguished patients with renal cell carcinoma from healthy donors with an area under the receiver-operating-characteristic curve of 0.88, and the same signal carried prognostic value, with higher serum Bacteroidia DNA associated with significantly poorer progression-free and overall survival during checkpoint-inhibitor immunotherapy (Uemura et al., 2023). This example illustrates both the diagnostic and prognostic potential of BEV-associated bacterial signatures and the field's current reliance on combinatorial, multi-marker indices rather than single-analyte tests to achieve clinically useful discrimination.

### 8.2 BEVs as vaccine platforms: from infectious disease to cancer immunotherapy

Among translational applications, vaccine development is the most established and clinically mature domain of BEV research. OMVs function as inherently self-adjuvanting nanostructures, presenting antigens within a native lipid context that promotes efficient uptake by antigen-presenting cells (APCs) and robust activation of innate immune pathways (Wen and Herman, 2024; Nie et al., 2025; Bhanu et al., 2025).

Recent advances in genetic and metabolic engineering have significantly expanded their applicability. By modifying producer strains to enrich desired antigens or attenuate pro-inflammatory components such as lipopolysaccharide (LPS), BEVs can be rationally engineered into highly tunable immunological platforms (Jeong et al., 2024; Luo et al., 2024; Asano-Inami et al., 2025; Chen et al., 2025). This has enabled their extension beyond infectious disease prophylaxis into cancer immunotherapy.

In this context, engineered BEVs derived from commensal gut bacteria are being actively explored as cancer vaccine platforms. These vesicles exploit intrinsic immunostimulatory properties to reprogram tumor-associated immune microenvironments and enhance anti-tumor immune responses (Chen et al., 2025; Lee et al., 2025; Ahn et al., 2025). Collectively, this positions BEVs at the interface of microbial engineering and precision immuno-oncology.

The clinical maturity of OMV-based vaccination is illustrated by outer membrane vesicle-containing serogroup B meningococcal vaccines, which have been in routine use for over a decade. A nationwide case–control study in Spain found complete 4CMenB vaccination to be 76% effective against invasive meningococcal disease of any serogroup and 71% effective against serogroup B disease specifically (Castilla et al., 2023). Beyond their intended target, these OMV-containing vaccines have also shown unanticipated cross-protection against the genetically related pathogen Neisseria gonorrhoeae: a systematic review and meta-analysis of case-control and cohort studies estimated a pooled vaccine effectiveness of approximately 35% against gonococcal infection (Wang et al., 2024). This cross-protective effect, first identified through population-level surveillance rather than by design, illustrates both the practical value and the still-incomplete mechanistic understanding of OMV-based immunization.

### 8.3 BEVs as therapeutic and drug-delivery platforms

Therapeutic applications of BEVs span both naturally derived and engineered modalities.

Native vesicles from beneficial taxa such as *Akkermansia muciniphila, Bifidobacterium*, and other commensals act as postbiotic effectors, recapitulating key immunomodulatory and barrier-protective functions of live probiotics while avoiding risks associated with bacterial colonization or systemic infection (Zhang et al., 2025; Han et al., 2024; Kozakiewicz et al., 2025; Chelakkot et al., 2018).

Beyond native functionality, BEVs are increasingly being repurposed as engineered delivery systems. Recent studies have demonstrated successful loading of vesicles with antimicrobial enzymes, including endolysins, enabling targeted elimination of pathogenic bacteria such as *Staphylococcus aureus*. These engineered constructs illustrate the emergence of BEVs as biologically derived nanotherapeutics with programmable antimicrobial activity (Shi et al., 2025; Pirolli et al., 2025a).

More broadly, advances in surface engineering and genetic programming have enabled the design of BEVs with enhanced targeting specificity and customizable cargo profiles, including chemotherapeutics and regulatory RNA species. These developments establish BEVs as adaptable nanocarriers for precision medicine applications spanning infectious, inflammatory, and malignant diseases (Xu et al., 2026; Ahn et al., 2025; Roier et al., 2016).

A recent example illustrates the extent to which OMVs can now be engineered as multifunctional therapeutic adjuncts rather than single-purpose carriers. In a chimeric antigen receptor (CAR) T-cell platform, OMVs were simultaneously modified to display an anti-PD-L1 single-chain antibody, to counteract checkpoint-mediated exhaustion, and to deliver a plasmid encoding the CAR target antigen, driving transient antigen expression on otherwise antigen-negative tumor cells. In immunocompetent mice bearing antigen-heterogeneous or antigen-negative breast and liver tumors, this combined platform substantially reduced tumor burden and prolonged survival relative to CAR-T therapy alone, and long-term rechallenge experiments indicated durable antitumor memory without evidence of off-target toxicity in non-tumor tissues (Li X. et al., 2026). Such multi-engineered constructs highlight the capacity of BEVs to simultaneously address several independent barriers to cellular immunotherapy within a single, modular delivery platform.

### 8.4 Opportunities and bottlenecks in clinical translation

Despite rapid conceptual and technological progress, BEVs remain a technically demanding translational platform. Major limitations include intrinsic heterogeneity, batch-to-batch variability, and the absence of standardized large-scale production systems (De Langhe et al., 2024; Welsh et al., 2024; Wang et al., 2026).

From a safety perspective, Gram-negative BEVs present a particular challenge due to their endotoxin content, necessitating careful calibration between immunostimulatory efficacy and reactogenicity (Wen and Herman, 2024; Nie et al., 2025; Bhanu et al., 2025). In parallel, key pharmacological parameters including biodistribution, circulation half-life, tissue tropism, and clearance mechanisms remain incompletely defined, limiting predictive translational modeling and clinical optimization (Luo et al., 2024; Asano-Inami et al., 2025).

Addressing these challenges will require integration of scalable manufacturing pipelines, standardized characterization frameworks, and systematic *in vivo* pharmacokinetic studies.

### 8.5 Outlook for clinical translation

BEVs represent a highly promising yet complex platform at the intersection of microbiology, immunology, and nanomedicine. Diagnostic applications are likely to achieve clinical translation first, particularly in oncology and inflammatory diseases where microbial dysbiosis plays a mechanistic role. In parallel, ongoing advances in vesicle engineering, safety optimization, and manufacturing standardization are expected to accelerate the development of BEV-based vaccines and therapeutics toward clinically deployable nanomedicine platforms (De Langhe et al., 2024; Zou et al., 2025; Wang et al., 2026; Asano-Inami et al., 2025).

## 9 Future perspectives and outstanding questions

Despite substantial advances in bacterial extracellular vesicle (BEV) research, the field remains at a critical juncture in which key conceptual, methodological, and translational uncertainties persist. Although BEVs are now widely recognized as active biological entities rather than passive metabolic by-products, core mechanistic claims including selective cargo loading, systemic signaling capacity, and causal involvement in disease remain incompletely resolved and require more rigorous experimental validation (De Langhe et al., 2024; Toyofuku et al., 2023; Wang et al., 2026).

### 9.1 Resolving vesicle heterogeneity: beyond bulk analyses

A major limitation in current BEV research is the intrinsic heterogeneity of vesicle populations. Most studies rely on bulk isolation strategies that obscure the presence and functional contribution of distinct vesicle subtypes (De Langhe et al., 2024; Toyofuku et al., 2023; Wang et al., 2026). Consequently, biologically and mechanistically divergent populations are frequently analyzed as a single functional entity.

Future work must prioritize subpopulation-level resolution, particularly discrimination between canonical outer membrane vesicles (OMVs) and lysis-associated outer–inner membrane vesicles (OIMVs) within the same strain. Achieving this will require integration of high-resolution density fractionation, nanoscale flow cytometry, and emerging single-vesicle multi-omics approaches. Such methodologies will be essential for linking biogenetic origin with functional output and host interaction specificity (De Langhe et al., 2024; Kulp and Kuehn, 2010; Wang et al., 2026).

### 9.2 From association to causation: the driver–passenger problem

As associations between BEVs and complex diseases, including inflammatory bowel disease (IBD), colorectal cancer (CRC), and neurodegenerative disorders continue to accumulate, a central unresolved issue is causality. It remains unclear whether disease-associated BEVs act as primary drivers of pathogenesis, secondary amplifiers of inflammation, or passive reflections of underlying dysbiosis (Sun et al., 2023; Chronopoulos and Kalluri, 2020; Mehmood and Danaf, 2025).

Resolving this “driver–passenger” problem will require a transition from correlative to mechanistic frameworks. This includes strain-specific vesicle engineering, longitudinal human cohort studies, and rigorous gain- and loss-of-function experimental designs. Only through such approaches can BEVs be definitively classified as causal effectors vs. biomarkers of disease state.

### 9.3 *In vivo* dynamics and tissue trafficking

A significant portion of current knowledge is derived from static *in vitro* systems that do not capture the dynamic and spatial complexity of BEV behavior *in vivo*. Critical parameters, including circulation half-life, biodistribution, tissue tropism, and barrier translocation capacity toward distal organs such as the liver and brain, remain insufficiently characterized (Jang et al., 2015; Kozakiewicz et al., 2025; Pawar et al., 2025).

Future studies should prioritize physiologically relevant tracking methodologies, including isotopic labeling, real-time *in vivo* imaging, and advanced organoid or organ-on-chip systems. These approaches are necessary to reconstruct the true spatiotemporal dynamics of BEV distribution across biological compartments.

### 9.4 Ecological realism in polymicrobial contexts

BEVs operate within highly complex microbial ecosystems characterized by interspecies competition, cooperation, and fluctuating environmental constraints. However, most mechanistic studies are conducted in simplified monoculture systems that fail to recapitulate the ecological and physicochemical complexity of natural microbiomes (Mashburn and Whiteley, 2005; Jeong et al., 2024; Kawagishi et al., 2025).

Future research must therefore incorporate polymicrobial biofilms and host-associated microbial consortia to better approximate *in vivo* conditions. In particular, the influence of nutrient gradients, host-derived factors, and interspecies signaling on vesicle production, stability, and functional persistence remains a critical and underexplored dimension.

### 9.5 Standardization as a prerequisite for clinical translation

A major translational bottleneck remains the lack of standardized protocols for BEV isolation, purification, characterization, and functional assessment. Clinical deployment of BEV-based diagnostics, vaccines, and engineered therapeutics depends on reproducible manufacturing pipelines and harmonized quality-control frameworks.

For engineered vesicle systems, including advanced delivery platforms, an additional challenge lies in balancing enhanced targeting or immunogenicity with safety, reactogenicity, and batch-to-batch consistency. These parameters must be tightly controlled to meet regulatory standards for clinical translation (Zou et al., 2025; Asano-Inami et al., 2025).

### 9.6 Outlook: toward a unified biological interface

The future of BEV research lies in the convergence of microbiology, immunology, and nanomedicine into a unified conceptual framework. Rather than being viewed as niche microbial by-products, BEVs should be recognized as fundamental biological interfaces that mediate information exchange between microbial physiology and multicellular host systems (De Langhe et al., 2024; Qian et al., 2025; Marquez-Paradas et al., 2025).

Progress in this field will depend on simultaneous advances in methodological rigor, mechanistic resolution, and ecological realism. With these developments, BEV research is expected to transition from fragmented descriptive observations toward a coherent, predictive, and clinically actionable framework underpinning next-generation microbiome-based diagnostics and precision therapeutics.

Bacterial extracellular vesicles (BEVs) have fundamentally reshaped our understanding of microbial communication, transitioning from historically perceived metabolic by-products into central, information-rich mediators of bacterial physiology and inter-kingdom signaling. Rather than passive remnants of envelope turnover, these nanoscale, lipid-bilayer–enclosed entities function as organized delivery platforms capable of transporting diverse molecular cargo, including proteins, lipids, nucleic acids, and small metabolites across complex polymicrobial environments and into distal host tissues (De Langhe et al., 2024; Qian et al., 2025; Mashburn and Whiteley, 2005).

By overcoming the spatial constraints of diffusion and direct cell-to-cell contact, BEVs extend the functional reach of the bacterial secretome and orchestrate key processes such as quorum sensing, biofilm dynamics, horizontal gene transfer, and systemic immune modulation (Mashburn and Whiteley, 2005; Nagasawa et al., 2025; Kawagishi et al., 2025; Nie et al., 2025; Marquez-Paradas et al., 2025).

A central conceptual advance in recent literature is the recognition of BEV functional duality across health and disease. In pathogenic contexts, BEVs act as highly efficient vectors of virulence, inflammation, and host manipulation, contributing to disease progression across multiple organ systems, including the gastrointestinal tract, liver, vasculature, and tumor microenvironments. In contrast, commensal- and probiotic-derived vesicles function as mediators of immune tolerance, epithelial protection, and host–microbiota mutualism (Nie et al., 2025; Han et al., 2024; Zheng et al., 2024; Tsubaki et al., 2026). This duality highlights a key principle: BEVs are not inherently beneficial or pathogenic, but context-dependent biological entities whose functional output is determined by microbial origin, cargo composition, and the physiological state of both donor and recipient systems.

Despite this conceptual maturation, the translational potential of BEVs remains constrained by unresolved methodological and analytical challenges. Vesicle heterogeneity, together with the lack of standardized isolation, purification, and characterization frameworks, continues to limit reproducibility and mechanistic interpretability across studies (De Langhe et al., 2024; Welsh et al., 2024; Wang et al., 2026). Addressing these limitations is not merely technical but fundamental to field advancement, particularly as BEVs transition from experimental constructs to clinically relevant platforms. In this context, integration of high-resolution analytical technologies with rigorous standardization will be essential for ensuring reliable functional attribution and translational scalability (Zou et al., 2025; Asano-Inami et al., 2025; Chen et al., 2025; Lee et al., 2025; Ahn et al., 2025; Shi et al., 2025; Pirolli et al., 2025a).

In conclusion, BEVs represent a foundational biological interface at the intersection of microbial ecology and host physiology. They function as dynamic, autonomous units of biological information that regulate microbial social behavior, environmental adaptation, and host interaction, ultimately shaping outcomes ranging from homeostatic balance to disease pathogenesis (De Langhe et al., 2024; Qian et al., 2025; Melo-Marques et al., 2024; Marquez-Paradas et al., 2025). As the field progresses toward a more mechanistically resolved and methodologically standardized framework, BEVs are poised to emerge not only as a unifying concept in microbiome biology but also as a transformative platform for next-generation diagnostics, vaccines, and precision therapeutics (Table 3).

**Table 3:** Outstanding questions and future research priorities in BEV research.

Research area	Current limitation	Future direction
Vesicle heterogeneity	Bulk analyses mask subpopulations	Single-vesicle omics
Cargo loading	Mechanisms poorly understood	Genetic and molecular mapping
*In vivo* trafficking	Limited biodistribution data	Advanced imaging and tracking
Clinical translation	Lack of standardization	Harmonized protocols
Biomarker development	Reproducibility issues	Multicenter validation studies

## 10 Conclusion

BEVs represent a biologically meaningful link between microbial communities and host systems. They act as carriers of molecular information that shape microbial interactions, immune responses, and systemic physiology.

Their functional effects are not fixed but depend on bacterial origin, cargo composition, and the surrounding biological environment. This variability explains why BEVs can contribute to both homeostasis and disease within the same biological framework.

Although interest in their clinical application is increasing, translation remains limited by methodological variability, particularly in isolation and characterization approaches. These issues continue to represent major barriers to reproducibility and cross-study comparability.

Future progress will depend on improving methodological standardization and resolving how distinct vesicle populations behave *in vivo*. Addressing these gaps will be essential for moving BEV research from descriptive observations toward clinically actionable applications in diagnostics and therapy.
